# Tourette syndrome research highlights from 2021

**DOI:** 10.12688/f1000research.122708.2

**Published:** 2022-11-01

**Authors:** Andreas Hartmann, Per Andrén, Cyril Atkinson-Clement, Virginie Czernecki, Cécile Delorme, Nanette Marinette Debes, Natalia Szejko, Keisuke Ueda, Kevin Black

**Affiliations:** 1 Department of Neurology, APHP, Sorbonne University, Hôpital de la Pitié-Salpêtrière, Paris, 75013, France; 2 Department of Clinical Neuroscience, Karolinska Institutet and Stockholm Health Care Services, Stockholm, Sweden; 3 Paris Brain Institute (ICM), Sorbonne Université, Inserm, CNRS, APHP, Paris, 75013, France; 4 Department of Child Neurology, Herlev University Hospital, Copenhagen, Denmark; 5 Department of Neurology, Medical University of Warsaw, Warsaw, Poland; 6 Department of Psychiatry, Neurology, Radiology and Neuroscience, Washington University in St. Louis, Saint Louis, Missouri, USA

**Keywords:** Tics, Tourette syndrome, 2021

## Abstract

We summarize selected research reports from 2021 relevant to Tourette syndrome that the authors consider most important or interesting. The authors welcome article suggestions and thoughtful feedback from readers.

## Introduction

This article is meant to disseminate recent scientific progress on Gilles de la Tourette Syndrome (TS). This is the eighth annual update for the Tics collection on F1000Research ( https://f1000research.com/collections/tics). The authors eagerly invite article suggestions for the 2022 highlights article at https://www.authorea.com/554771/.

## Methods

We searched PubMed using the search strategy (“Tic Disorders”[MeSH] OR Tourette NOT Tourette [AU]) AND 2021[PDAT] NOT 1950:2020[PDAT]. On 10 January 2022 this search returned 306 citations, available at this link. Colleagues also recommended articles, and we attended selected medical conferences. We selected material for this review subjectively, guided by our judgment of possible future impact on the field.

## Results

### Phenomenology and natural history

*Definition*

A very compelling data analysis and review argues for the conclusion that persistent (chronic) motor or vocal tic disorder (PMVT) and Tourette Disorder (TS) are the same illness, or at least that “PMVT and TS occur along a clinical spectrum in which TS is a more severe and PMVT a less severe manifestation of a continuous neurodevelopmental tic spectrum disorder.” ^1^

The European clinical guidelines for Tourette syndrome were updated (version 2.0) with regard to assessment of tic disorders. ^2^ As an introduction to these updated guidelines, it is well worth reading the summary statement ^3^ and the patients’ perspectives. ^4^

*Epidemiology*

When it comes to sex differences, a review by Garris and Quigg described that although TS is more common in boys, tics in girls tend to be more persistent. ^5^ Also, comorbidity differed, with ADHD being more common in boys and mood and anxiety disorders more common in girls. This topic was also explored by the European Multicentre Tics in Children Studies (EMTICS) group who showed that males, in general, had more severe symptoms than females with the exception of emotional problems. Since male symptoms seem more predominant at a younger age, their diagnosis might be facilitated compared to females. ^6^

As for the course of tics, another EMTICS study assessed clinical precursors of tics by comparing 61 high-risk children who did develop tics in a seven-year follow-up period with 126 high-risk children who did not develop tics. Male sex, severity of conduct problems, autism spectrum disorder (ASD), compulsions, and emotional problems were shown to be precursors to tic occurrence. ^7^

A study by Gromark and colleagues also tackled the topic of disease prognosis and course, but in a cohort of individuals with Pediatric Acute-onset Neuropsychiatric Syndrome (PANS). ^8^ Over a median of 3.3 years after the diagnosis, most symptoms improved substantially and only 35% of children had a chronic, non-remitting course. These results suggest a generally positive outcome for children diagnosed with PANS, and interestingly may not differ substantially from the outcome in recent studies in non-PANS children, including the population of Provisional Tic Disorder. ^9^^,^^10^

Another important report regarding treatment of tics comes from China. ^11^ Demographic and prescription data regarding tic disorder were extracted from the electronic medical records database of Beijing Children’s Hospital from 2018 to 2020. The authors paid special attention to treatment choices. All in all, 20,417 patients were included and 28.1% (n=5028) of them were treatment-free. In contrast, 70% received anti-tic medication. In line with reports from other countries, in children less than six years of age, clonidine was the most frequently employed medication (in particular, clonidine adhesive patches, CAP). Another treatment option commonly used in this group was traditional Chinese medication (TCM). As patients were older, the use of antipsychotics was growing. As for the number of medications prescribed, 22% (n=3389) were treated with at least two compounds, with the most common combination being tiapride and TCM (33.7%), followed by CAP and TCM (22.1%).

Regarding reports on particular types of tics, Kaczyńska and Janik reported their experience with tonic (isometric) tics in 241 consecutive TS patients (including 153 children). ^12^ Tensing of the abdomen was the most common localization, followed by the neck and upper extremities. Tonic tics were common, occurred early in the course of the disease, and were associated with greater number, severity and complexity of tics. The same authors covered the topic of blocking tics in the setting of a one-time registration study including 195 consecutive TS patients. Blocking tics were found frequently (37.4%) and were early associated with more severe disease phenotype. ^13^

The controversial topic of cognitive tic-like phenomena was studied by Janik and colleagues. ^14^ They included 227 consecutive TS patients. Cognitive tic-like phenomena were defined as brief and sudden involuntary thoughts. However, since they are not accompanied by anxiety, they are not considered part of the OCD spectrum. These phenomena occurred in 15% of patients and were correlated with older age, increased tic severity and anxiety disorder.

David Mataix-Cols’s lab reported a national registry study of cervical spine injuries in TS. Risk of vascular and nonvascular C-spine injuries were 38% to 57% higher compared to controls, without a significant difference by sex. This epidemiological study confirms previous case reports and suggests that, although most TS patients do not injure their spine, doctors should carefully monitor patients with severe disease or nuchal symptoms, and have a low threshold for intervention, since these injuries can be serious and persistent.

An interesting case series described six patients whose driving capacity was impaired by their tics. This is a highly relevant and rather frequent topic of discussion in TS clinics, and relies more on common sense than established guidelines, as in epilepsy. On the same topic, a survey study with 228 adult participants with self-reported chronic tic disorder (CTD) or TS showed that tics in most individuals did not influence driving. ^15^ However, reported tic severity was significantly higher among those without a driver’s license compared to those with a driver’s license, and tic severity was indicated as cause of not having a driver’s license in 60.7%.

*Comorbidity*

Zinna and colleagues performed a retrospective, naturalistic study of all children with CTD and TS admitted to an inpatient mental health unit during a 10-year period. 19.7% of the children needing inpatient treatment had comorbid CTD/TS, especially with obsessive compulsive disorder (OCD), intellectual disability, and autism spectrum disorder. ^16^ Moreover, presence of these co-existing disorders was associated with longer admissions. Interestingly, no association with CTD/TS and attention deficit hyperactivity disorder (ADHD) was found. Park and colleagues performed a retrospective review of medical records of 119 pediatric patients diagnosed with TS. The mean age at onset of tics was 6.9 years and mean age at diagnosis was 8 years. 31.1% had at least one comorbid neuropsychiatric disorder. ^17^ Tic severity was associated with shorter time to diagnosis and greater functional impairment.

Vermilion and colleagues compared anxiety symptom profile in youth with tic disorders with a community control group and a group of youths with clinically significant anxiety who were seeking anxiety treatment. ^18^ A weak, but significant, association between severity of tics and anxiety was found. Youths with tic disorders had similar anxiety severity as youths with clinically significant anxiety, and significantly higher anxiety severity than healthy controls. Separation anxiety severity in youths with tic disorders was significantly higher than in the group with anxiety.

Another Swedish registry study of over 10 million people found a 6.7-fold increased prevalence of insomnia in the 5,877 who had a recorded diagnosis of TS/CTD, after adjusting for demographics and somatic illness. ^19^ Excluding people with ADHD, ASD, or a sibling with TS/CTD reduced the odds ratio somewhat, suggesting that part of the risk is attributable to familial factors or these two comorbid diagnoses. Medication for ADHD also increased the likelihood of insomnia. These results suggest that assessment for sleep quality is important in managing TS. The association could be partly explained by ADHD, ADHD medication, familial factors, and neurodevelopmental comorbidities. This was confirmed by the findings in the meta-analysis by Keenan and colleagues, which included polysomnography studies in three groups of patients: CTD-only, ADHD-only, and CTD+ADHD. The CTD+ADHD group had most sleep difficulties, but both in CTD-only and CTD+ADHD group lower sleep efficiency and higher sleep onset latency were seen. ^20^

The etiology of anger outbursts and aggressive symptoms in patients with TS may be multifactorial and influenced by severity of illness and psychosocial factors. ^21^ In relation to this topic, it is also worth mentioning a small case series published by Kurvits and colleagues that explored the topic of allo-aggression and presented helpful tips to distinguish between allo-aggressive behaviors and tics. ^22^ The authors present cases of three patients with variety of complex repetitive behaviors and allo-aggression (e.g., sudden kicking, hitting, slapping and biting others, or pushing someone off a bike) which were mistaken for tics. An exhaustive clinical examination revealed the presence of red flag psychiatric symptomatology atypical for TS, such as blackouts or feeling of being possessed by different personalities.

A very interesting study by Vicario and colleagues ^23^ explored the topic of moral reasoning (MR) in TS. The hypothesis about altered MR in TS is based on altered social cognition and reduced cognitive empathy that has been found in TS. Comparing the results of variety of tests assessing MR between 21 TS patients and 21 healthy controls, the authors documented a greater tolerance of unethical behaviors in TS adolescents. However, the sample size was very small and further studies are clearly needed to validate these findings. Also, it will be important to assess in detail comorbidities that may influence moral reasoning.

As for other, non-psychiatric comorbidities, in a cohort of 201 patients with TS, 33.3% turned out to have comorbid non-tic movement disorders, particularly piano-playing movements (11%) and stereotypies (8%). ^24^ The most common etiology was drug-induced movements (6.0%) but in most cases no etiology was found and no association with severity of tics was found. The presence of connective tissue-altered conditions, like joint hypermobility, was shown to be associated with tic disorders. ^25^

*Premonitory urge and tic suppression*

Several studies have explored the topic of premonitory urge (PU) in TS. Ramsey and colleagues ^26^ investigated the nature of relationship between PU and disease severity in TS using structural equation modeling. This analysis revealed that higher levels of urge intolerance predicted greater levels of tic-related disability. Moreover, the association between urge intolerance and tic-related disability was more pronounced in adolescents with internalizing symptoms. Another study, by He and colleagues, ^27^ used magnetic resonance spectroscopy to examine the pattern of alterations in γ-aminobutyric acid (GABA) and glutamate neurotransmission in the following regions of the brain: the right primary sensorimotor cortex (SM1), supplementary motor area (SMA), and insular cortex. Interestingly, neither GABA+ nor glutamine levels were associated with tic diagnosis or severity. However, in children with TS, lower levels of SMA GABA+ were associated with more severe and more frequent premonitory urges. The authors concluded that GABAergic neurotransmission in the SMA area is involved in the development of PU. Schubert and colleagues ^28^ explored inter-individual differences in PU and urge-tic associations in 21 adult patients with TS. They demonstrated that, as expected, at the group level there was a positive association between PU and tic frequency and intensity. In addition, they demonstrated inter-individual differences in associations between urges and tics. The majority of participants (57-66%, depending on the measure that was used) demonstrated positive associations between tic severity and PU, but this was not the case for all patients and in two cases there was a negative association with tic occurrence and PU.

As for studies focused on tic suppression, Morand-Beaulieu and colleagues ^29^ investigated brain correlates of tic suppression. They applied high-density electroencephalography at rest and during tic suppression. Graph theoretical analyses showed a distinctive global network topology during tic suppression in comparison to rest. The authors complemented their analysis with network-based statistics and found a subnetwork of increased connectivity during tic suppression. The main regions of the brain involved in this network were right superior frontal gyrus and the left precuneus. Interestingly, there was a condition-by-age interaction, suggesting that with age brain circuits responsible for tic inhibition mature.

*Other*

Disease-related quality of life (QOL) in adults with TS at a subspecialty neurology movement disorders center did significantly correlate with current tic severity. ^30^ However, QOL was explained primarily by current *non-tic* symptom severity (in decreasing order of correlation strength: anxiety, ADHD, and OCD, followed by the Yale Global Tic Severity Scale [YGTSS] total tic score [TTS]). In a hierarchical regression analysis, the association of TTS with QOL was statistically significant only after removing the other variables.

Adults with TS (n=53, compared to 53 tic-free controls) had higher levels of food sensitivity and avoidance, and food neophobia was predicted by greater sensitivity to taste (gustatory hypersensitivity) in a study by Smith and colleagues. ^31^

### Etiology

*Genetics*

A genome-wide pathway analysis conducted by the TS/OCD working group of the Psychiatric Genomics Consortium used 1,285 cases with TS and 4,964 ancestry-matched controls. The study identified three gene sets as differing in TS: (i) ligand-gated ion channel signaling, (ii) lymphocytic (driven by variants in *FLT3*), and (iii) cell adhesion and trans-synaptic signaling sets. ^32^ These gene sets are in line with and reinforce previous hypotheses regarding the potential roles of neuroinflammation, GABA, and cell adhesion in the pathophysiology of TS.

A cross-disorder genome-wide association study (GWAS) on 93,294 individuals revealed genes that contribute shared genetic risk for TS in combination with ADHD, ASD, and OCD. ^33^ Regarding the TS-ADHD-ASD association, tissue specific analysis implicated the hypothalamus-pituitary-adrenal gland axis, in accordance with previous clinical studies implicating this system in multiple childhood-onset psychiatric traits, including TS and ADHD.

In a large (n=122) and densely affected pedigree of individuals with TS, 66 DNA samples were available and analyzed using different methods. ^34^ Overall, this family had a high load of common risk alleles for TS, suggesting that multiple common variants contribute more to risk than a few variants of strong effect.

Jones and colleagues ^35^ prospectively recruited 200 sequentially referred children with tic disorders/OCD, 100 autoimmune neurological controls, and 100 age-matched healthy controls. Using a structured interview, the maternal and family history of autoimmune diseases and other pro-inflammatory states was captured. Maternal blood and published Tourette brain transcriptomes were analyzed for overlapping enriched pathways. Overall, this fascinating study suggested that innate immune signaling may link maternal inflammation and childhood tics/OCD, and that targeting inflammation may represent a plausible therapeutic target in neurodevelopmental disorders, including TS. This line of argument was developed and expanded in two reviews on this topic by the same group. ^36^^,^^37^

In an analysis of genome-wide DNA methylation patterns in whole blood samples from 16 monozygotic twin pairs (eight discordant and six concordant for TS, plus two asymptomatic pairs), no site reached statistical significance, but a strong association was suggested with genes belonging to the mTOR pathway, previously implicated in a variety of neuropsychiatric disorders. ^38^

*Environmental risk factors*

The EMTICS study published its results testing the pediatric autoimmune neuropsychiatric disorders associated with streptococcal infection (PANDAS) hypothesis. ^39^ This study followed 715 children with TS/CTD for an average of 16 months. Tic severity was measured thrice annually, and infection with group A beta-hemolytic streptococcus (GAS) was monitored carefully using pharyngeal swabs and serologic testing. The study identified 405 tic exacerbations in 308 participants, but these exacerbations did not correspond temporally to GAS exposure. The authors conclude that their “study does not support GAS exposures as contributing factors for tic exacerbations in children with CTD. Specific work-up or active management of GAS infections is unlikely to help modifying the course of tics in CTD and is therefore not recommended.” ^39^ These results and conclusion support those of previous studies. ^40^

Ayubi and colleagues reviewed data from previously published epidemiological studies and concluded that maternal smoking during pregnancy raised the risk of TS in their children by 35%. ^41^

This year brought widespread attention to social contagion as a possible cause of sudden-onset tic-like symptoms in adolescents. Such a phenomenon had been described previously, for instance ten years ago in a group of high school students in the Rochester, New York, area, ^42^^,^^43^ but in 2020, Müller-Vahl and German colleagues reported a rush of cases among patients who did not know each other in person but had watched videos from the same social media personality and showed his same symptoms. ^44^ A report from London ^45^ reported a similar phenomenon and was picked up by numerous media outlets (a Google search on 13 November 2021 for “functional tic-like behaviors, TikTok, social media” returned about 100 relevant results). Vera and colleagues carefully examined popular ‘Tourette’ TikTok videos with unusual symptoms and described a number of features of these videos that are highly unusual for TS. ^46^ These features include aggression (in 19% of these videos), self-injurious behaviors (28%), coprophenomena (over half), long phrases (more than three words, 46%), throwing objects (22%), and very strong influence by the environment (over half). Senior clinicians viewing these videos rated them on a Likert scale of one (“All the tics are typical of TS”) to five (“None of the tics are typical of TS”). A group from Lübeck and Dresden directly compared demographic and clinical variables of 13 cases starting dramatically after social media exposure to 13 TS patients similar in age and sex. ^47^ A number of features were seen only in the post-social-media patients (i.e., 100% specific), including abrupt symptom onset, first symptom being complex, primarily slow and tonic movements, mostly trunk or extremities, a rapidly varying repertoire of symptoms, symptom deterioration in the presence of others, lack of spontaneous symptom fluctuations over the course of weeks to months, a symptom involving goal-directed movement (e.g., directed at another person), and dramatic context dependence. Symptoms continuing unabated throughout the examination was nearly as specific (13 of 13, *vs.* 1 of 10 with TS). Copropraxia was present in over half, as was echolalia, and symptom onset was significantly later (mean 15.3 years, *vs.* 5.2 years for TS). For all these reasons, most experts concluded that these presentations comprised a functional movement disorder rather than a variant expression of TS.

One concern raised early in this FND epidemic was that if the very features clinicians used to define FND patients turned out to be more common in their FND patients, one has not proved anything. Pringsheim and colleagues authored one of the first prospective studies that fully addresses that concern. ^48^ They reported on a prospective cohort of 290 children with tic-like phenomenology seen at Calgary. Twenty of these had rapid onset of clinically problematic tics. Crucially, these patients were *not* defined by clinical judgment of FND, so the results cannot be circular. These 20 rapid-onset patients differed substantially from the remaining 270 children with a (typical) primary tic disorder. They were an average of 7.5 years older at onset, 3.8 years older at the first visit to the center, had more severe tics (YGTSS total tic score 14.9 points higher, and impairment score 12.8 points higher, on average), and had more severe anxiety and depression scores (all of these *p* < .0001). Several dichotomous features were significantly more common in the rapid-onset group, though specificity for rapid-onset group membership was variable: 56% for ADHD, 84% for female sex, 81% for an anxiety disorder, and 96% for a depression diagnosis. The Tourette Association of America convened an international working group that provided a summary and recommendations for assessment and care ^49^; another helpful resource for patients is available at NeuroSymptoms.org. ^50^

### Pathophysiology

*Animal models*

Benzodiazepines and ethanol are among the well-known positive allosteric modulators (PAMs) of GABA-A receptors. A PAM highly selective for GABA-A receptors containing α6 subunits showed efficacy in the D1CT-7 transgenic mouse model of tics. ^51^ Dopamine antagonists showed similar effects but induced catalepsy, whereas this PAM did not. These results suggest a strategy for future human studies, including for treatment of tic disorders; benzodiazepines are widely prescribed for TS but there is a paucity of controlled data supporting their efficacy for tics.

Likely the most relevant and best established rodent models for tics have been developed by the group of Izhar Bar-Gad. In this paper, they provide a comprehensive overview of their work so far. ^52^

### Electrophysiology

*Electroencephalography*

Several studies using electroencephalography (EEG) to elucidate the pathophysiology of tic disorders were published in 2021. An EEG study of lateral readiness potentials, a measure of activation and preparation of responses occurring in motor cortical areas, showed that action integration *per se* was normal in patients with TS, suggesting that TS is not only a movement disorder. ^53^ EEG studies characterizing oscillatory activity and functional connectivity revealed that children with tics exhibited abnormal activation and communication patterns within the frontal-parietal lobe network during cognitive inhibition ^54^ and that children with TS suppressed tics through a distributed brain circuit of cortical regions. ^29^ In an EEG study of TS patients and controls, movement-related EEG (i.e., mu- and beta-band oscillations) was examined just before voluntary movements and tics were performed. ^55^ Mu and beta oscillations were not observed before tics, suggesting that a network of large brain regions (insular cortex, cingulate cortex, basal ganglia, cerebellum, etc.) is involved in the development of tics. In the same study, beta-band desynchronization occurred when TS patients initiated voluntary movements and, in contrast to healthy controls, no desynchronization of mu-band oscillations was observed during the execution of voluntary movements, which was interpreted as a physiological inhibition impairment in TS. Another study using EEG was conducted to calculate scale-free activity (1/ *f* neural noise) based on the theory that tics are behavioral surplus, or “motor noise”. ^56^ Although task-related 1/ *f* noise and high-frequency band aperiodic activity were found to be increased in patients with TS during sensory-motor processing, there was no evidence that scale-free and aperiodic activity was related to the process of tic development. The authors suggested that increased 1/ *f* noise and aperiodic activity are not directly related to tics, but more likely related to a new aspect of TS. Systematic review and meta-analysis investigating the electrophysiological correlates of performance monitoring showed that error-related negativity on EEG showed significantly increased amplitude in TS patients. ^57^ However, this was based on only five studies and there was a high degree of heterogeneity between studies.

*Neuroimaging studies*

In 2021, two voxel-based morphometry (VBM) meta-analyses were published. ^58^^,^^59^ Their objectives were the same (i.e., comparing TS patients to healthy controls), but they used different approaches and found different results. The first ^59^ selected 10 studies and used a voxel-wise meta-analysis called seed-based *d* mapping. The second ^58^ selected six studies and used the more classical Activation Likelihood Estimation (ALE) method. They both reported increased gray matter (GM) volume in the thalamus and the putamen and decreased GM in the postcentral gyrus. Surprisingly, their remaining results differed and can therefore be considered more controversial: increased GM volume in the cerebellum (vermis III), in limbic areas (striatum, insula, and hypothalamus) and in sensorimotor regions (pre- and post-central gyri); decreased GM volume in frontal (inferior and medial parts and rolandic operculum), temporal (superior temporal gyrus) and parietal cortices (supramarginal gyrus) as well as cingulate gyrus (anterior and posterior parts).

The anterior cingulate cortex (ACC) is largely suspected to play an important role in TS. A study using both VBM and structural covariance network mapping focused on this region. ^60^ This study found, first, decreased GM volume within the ACC and, second, increased structural covariance between the ACC and the motor parts of the cerebellum, the inferior frontal cortex and the posterior cingulate cortex.

Interesting functional magnetic resonance imaging (fMRI) studies in TS were also published last year. One used multivariate analysis with a support vector machine (SVM) to distinguish TD patients from healthy controls. ^61^ This classification algorithm reached a correct general accuracy of 67% to distinguish the two groups, especially on the basis of resting-state fMRI data within the striatum, the fronto-parietal cortex, and the cerebellum. In addition, the authors distinguished medicated and unmedicated patients with an accuracy of 69% based on the activity of the striatum, the insular and cerebellar networks. These results and previous similar work ^62^ show that resting state functional imaging contains information relevant to distinguishing diagnosis and other clinical features in TS.

From a cognitive standpoint, the neural networks that underlie urge inhibition were assessed in TS patients with obsessive-compulsive symptoms during a task involving blink suppression while viewing emotional (angry and neutral) faces. ^63^ The authors found that, compared to healthy controls, patients had higher activity in the superior temporal gyrus and the middle cingulate cortex. In addition, by decomposing the task, they found that (1) tic severity was related to higher activity during angry faces trials in comparison to neutral faces; (2) premonitory urge severity was related to higher activity in the hippocampus, middle temporal gyrus, thalamus, and caudate nucleus; and that (3) blink inhibition was associated with decreased activity in both the thalamus and the insular cortex.

Another fMRI study focused on decisional impulsivity by using a delay-discounting task involving choosing between a small immediate reward or a larger delayed reward. ^64^ While they found no abnormal decisions in patients in comparison to a group of healthy controls, the authors identified a subgroup of patients with higher impulsivity. This group was also characterized by a higher burden of impulse-control disorders and a higher level of general impulsivity. In this group, reward discounting was related to brain activity in a network comprising the orbito-frontal, cingulate, pre-supplementary motor area, temporal and insular cortices, as well as ventral striatum and hippocampus. However, the most interesting result was that a greater connectivity of pre-supplementary motor area with anterior insular cortex predicted both steeper reward discounting and more severe tics. This suggests a relation between cognitive (decision-making) and motor (tics) impulsivity in this subgroup of patients.

*Neuropsychology*

Sturm and colleagues investigated inhibitory control abilities in 24 TS patients, 139 ADHD patients, 19 TD+ADHD patients, and 59 typically developing controls aged nine to 14 years. ^65^ Inhibitory control was measured using common neurocognitive tasks [Attentional Network Task (ANT), Stop Signal Task (SST), Delis-Kaplan Stroop task, and Go-Nogo task]. Surprisingly, their results did not confirm the inhibitory control deficit that had previously been found in TS or TS+ADHD patients (reviewed by Morand-Beaulieu et al. ^66^). But the originality of this paper was to evaluate how inhibitory control deficit, tic severity (YGTSS), ADHD symptoms severity (by the Strengths and Weaknesses of Attention-Deficit/Hyperactivity symptoms and Normal behaviors scale, SWAN), and subjective tic suppressibility (item 10 on the Premonitory Urge for Tics scale, PUTS), contribute to objective tic suppressibility (tic suppression paradigm ^67^). The authors found that the only predictor of performance in the tic suppression paradigm was subjective tic suppressibility, and that inhibitory control deficit, tics, and ADHD severity had no effect. As objective tic suppression is known to be a potential mechanism of behavioral treatment response for TS patients, these findings have interesting clinical implications.

*Tic assessment*

An important study by Haas and colleagues evaluated the psychometric properties of the gold standard for tic assessment, namely the Yale Global Tic Severity Scale (YGTSS). The authors used a subsample of the EMTICS including 706 children and adolescents with a chronic tic disorder, and investigated convergent, discriminant, and factorial validity, as well as internal consistency, of the YGTSS. The YGTSS demonstrated acceptable psychometric quality. Nevertheless, they also showed that there is a need for further improvement of some items, for example, the global severity (total) score, indicating that separate use of the total tic score and the impairment rating is more beneficial. ^68^

Psychometric properties of the Chinese version of the YGTSS were explored by Wen and colleagues. ^69^ The authors included 367 children and adolescents with tic disorders and tested both reliability and validity. Overall, the Chinese version of YGTSS showed good psychometric properties. Similarly, Li and colleagues ^70^ tested the psychometric properties of another gold standard scale used in tic research, the Premonitory Urge for Tics scale (PUTS), in the Chinese population. No differences regarding PU manifestation were found in diverse age groups. The exploratory factor analysis of PUTS demonstrated the emergence of four primary factors. Finally, reliability and validity analyses indicated that the Chinese version of PUTS had good psychometric properties.

Many studies have focused on the development of new assessment tools for tic assessment, especially using new technologies. In one study by Eriguchi and colleagues, the authors tested a new method used to quantify the angular movements of neck tics using a compact gyroscope. The authors also investigated the clinical course of neck tics during a follow-up period of two years. Although the authors did not detect any changes in intensity and frequency of neck tics during the follow-up period, the gyroscope proved to be a valuable solution to monitor severe, healthy, or even life-threatening neck tics. ^71^ Another approach using new technologies was proposed by Cernera and colleagues who tested a sensor-based technology called ‘the human tic detector’ to detect and classify tics. ^72^ The authors recorded both surface electromyogram and acceleration data from 17 patients with TS when performing voluntary and involuntary movements (tics) using the modified Rush Video Tic Rating Scale (mRVTRS). In addition, they evaluated spectral properties of voluntary and tic movements with a sensor capturing the dominant tic and used a support vector machine (SVM) to detect and classify movements. It was demonstrated that wearable sensors can be used to distinguish between tics and voluntary movements and are comparable to expert consensus. Paulus and colleagues analyzed videos of 101 patients with TS and 109 healthy controls according to the Modified Rush Videotape Rating using a machine learning-based analysis. Severity of motor tics was shown to be the best predictor of a diagnosis of TS. Interestingly, vocal tics did not predict TS diagnosis, which might challenge the validity of the current diagnostic criteria for TS, since the presence of vocal tics has so far been a *conditio sine qua non* for TS.

One important analytical avenue used both for data analysis as well as development of tic assessment algorithms is artificial intelligence. This approach was used by Wu and colleagues, who attempted to find an automatic method for detecting tics to assist in diagnosis and evaluation. ^73^ Based on clinical data, the authors proposed a deep learning algorithm using both unsupervised and supervised learning and learning features from videos for tic motion detection. Taken together, the proposed models demonstrated satisfactory recognition between tics and non-tic movements, and the authors concluded that this methodology could be potentially useful in clinical care.

### Treatment

*Psychological interventions*

Treatment guidelines published by the American Academy of Neurology (AAN) in 2019 ^74^ and the European Society for the Study of Tourette Syndrome (ESSTS) in 2021 ^75^ recommend behavior therapy (BT) as the first-line intervention for TS/CTD. Out of several types of BT, recommendations are primarily for Habit Reversal Training (HRT) and its extended package Comprehensive Behavioral Intervention for Tics (CBIT), and secondarily for Exposure and Response Prevention (ERP).

Several research groups are currently investigating ways to make BT more accessible to healthcare seeking TS/CTD individuals, including various remote formats. In a British randomized controlled trial (RCT) by Hollis and colleagues, ^76^ referred to as the ORBIT study, 224 young TS/CTD individuals were randomized to therapist-supported, internet-delivered ERP (the previously piloted Swedish BIP TIC ERP program ^77^) or a therapist-supported, internet-delivered psychoeducation comparator. Participants in the ERP group improved on average 4.5 points (16%) on the primary outcome (tic severity as measured by the YGTSS-TTSS) from baseline to post-treatment, compared to 1.6 points (6%) in the comparator group, a significant interaction of group and time showing superiority for the BIP TIC ERP intervention. The ORBIT study is a significant contribution to the field, being the largest study of BT for TS/CTD to date and the first study to show ERP to be superior to an active control intervention. An ongoing similar Swedish RCT will further add to the evaluation of the BIP TIC ERP program. ^78^ Time will tell whether BIP TIC ERP will be implemented into regular healthcare.

The internet-delivered format has also recently been evaluated among adults. In a German RCT by Haas and colleagues *,* ^79^ 161 adult TS/CTD individuals were randomized to unsupported internet-delivered CBIT ( *n*=67), unsupported internet-delivered psychoeducation ( *n*=70), or face-to-face CBIT ( *n*=24). The primary comparison between the two internet-delivered interventions showed a trend towards superiority of internet-delivered CBIT in reducing tic severity (as measured by the YGTSS-TTSS) at the primary endpoint (post-treatment). This trend later evolved to a statistically significant superiority of internet-delivered CBIT at follow-ups three and six months post-treatment. Even though the tic severity improvements were lower than in RCTs of face-to-face CBIT, and the study experienced relatively high dropout rates, internet-delivered CBIT may be a cost-effective (i.e., no therapist support is required) and helpful treatment option for some TS/CTD individuals.

A few smaller studies have also investigated remote treatment formats. Viefhaus and colleagues ^80^ piloted a blended approach, where online therapist support *via* videoconferencing was added to a regular face-to-face HRT intervention. The aim was two-fold: to provide therapist support for homework directly and to reduce the need for travel to the clinic. The format was shown feasible in a case series of children and adolescents ( *N*=5). Reese and colleagues ^81^ conducted a pilot study ( *N*=8) of an online mindfulness-based intervention (self-help mixed with therapist-supported group sessions via videoconferencing) for adults with TS/CTD. The authors concluded that the intervention was feasible and acceptable, but that participant adherence to the mindfulness homework assignments was lower than expected. Individual improvements in YGTSS-TTSS were also modest. Further refinement and evaluation of this intervention is needed.

A traditional group format, without the online component, may also be an efficient way to save therapist resources and promote the dissemination of BT. Zimmerman-Brenner and colleagues ^82^ randomized 61 young TS/CTD individuals to group CBIT or group psychoeducation. The study showed somewhat mixed findings, with no significant between-group effect on the YGTSS-TTSS at the primary endpoint. Unexpectedly, tic severity increased in both groups between baseline and post-treatment, to later decrease at three-month follow-up. The temporary increase in symptoms seems to have been driven by an increase in vocal tic severity, potentially a side effect of the group format. Limitations included not including a power calculation nor performing intention-to-treat analyses.

During 2021, a few variations and extensions of previous studies of face-to-face CBIT were also conducted. In a small pilot study (N=6), Peterson and colleagues ^83^ investigated the specific effect of relaxation training as part of the CBIT package (alongside HRT and function-based interventions). The study concluded that relaxation training alone was not effective in reducing tic severity; rather, it should be included in conjunction with other CBIT elements.

Espil and colleagues ^84^ followed up young participants 11 years after receiving CBIT or supportive therapy and education (comparator) in the Piacentini and colleagues RCT from 2010. ^85^ This is the longest published prospective follow-up to date of TS/CTD individuals having received BT. The results showed a significant tic severity decrease across the sample during the follow-up period. Further, treatment responders to both interventions in the original study achieved at least partial tic remission during the follow-up period (a YGTSS-TTSS score <14), of which treatment responders in the CBIT group were more likely to achieve remission than treatment responders in the comparator. Despite limitations such as confounding TS/CTD interventions during the follow-up period and data loss, the study indicated good durability of the effects of BT.

Also based on previous RCTs of CBIT, ^86^ Essoe and colleagues ^87^ investigated the relation between homework adherence and treatment outcomes. The results showed that greater overall homework adherence predicted tic severity reductions and treatment response. Essoe and colleagues separately reviewed literature on the working mechanism of BT for TS/CTD. ^88^ This review showed that mechanisms appear to differ between youth and adults. Data primarily supported associative learning (e.g., positive reinforcement) and cognitive control (e.g., active tic inhibition) as mechanisms, while habituation may be a mechanism for adults only. Interpretation is made difficult by a mixture of laboratory and clinical studies, overall small sample sizes, and different ways to measure phenomena such as premonitory urge intensity.

*Medication*

The European clinical guidelines for Tourette syndrome were updated (version 2.0) with regard to pharmacological treatments. ^89^

A number of randomized controlled studies have investigated the potential efficacy of new (extended release) VMAT2 inhibitors for the treatment of tics. To date, the mother compound, tetrabenazine, has been used largely off label for tic treatment in the absence of evidence derived from high quality clinical studies. Alas, both deutetrabenazine ^90^^,^^91^ and valbenazine failed to meet primary endpoints in their respective studies. ^92^

An interesting case series described six children or adolescents with aggressive behaviors in whom lurasidone, a novel antipsychotic, was added to either aripiprazole or risperidone; the addition of lurasidone appeared to diminish these symptoms with good tolerability. ^93^

Interest in cannabis (and, more generally, the cannabinoid system) in the treatment of tics is ongoing. Monoacylglycerol lipase inhibition to increase levels of 2-arachidonoylglycerol (2-AG), an endocannabinoid, was tested in a 12-week, multicenter, randomized, placebo-controlled, double-blind clinical trial using a compound called Lu AG06466 (formerly known as ABX-1431). This was a phase two trial in adult patients with TS. There were no significant differences on the YGTSS between groups, nor for other endpoints assessing tic severity, premonitory urges, quality of life, and common psychiatric comorbidities. Further development of the compound for tics/TS was abandoned. ^94^ In contrast, encouraging results were obtained by Bloch and colleagues for THX-110, a combination of Δ ^9^-tetrahydracannabinol (Δ ^9^-THC) and palmitoylethanolamide (PEA) in a 12-week uncontrolled trial in 16 adult patients with TS. ^95^ Tic symptoms improved on average by more than 20% (=7-point decrease in the YGTSS score), and 12 of the 16 participants elected to continue to the extension phase. However, tolerability was slightly tricky and, of course, these results now require validation in randomized double-blind placebo-controlled trials.

Finally, another treatment target in TS might be the serotonergic system. Pimavanserin, a serotonin 2A receptor inverse agonist and antagonist, was tested in an open label trial over eight weeks in 10 adult patients with TS. A small but noteworthy decrease in tic severity (-12%) was noted on the YGTSS-TTS, as well as improvements on scales assessing global clinical impression and quality of life. Here too, larger, placebo-controlled trials are warranted. ^96^

*Neurosurgery*

Several important papers have been published in 2021, which provide interesting insights for clinical management of these patients and also for the better understanding of the neural bases and brain targets involved in deep brain stimulation (DBS) effects.

Two important guidelines papers were published this year, which may be useful to refine inclusion criteria for DBS in TS patients. Szejko and colleagues published a revised version of the ESSTS guidelines on DBS based on data from the literature and the International Tourette DBS registry and database. Important takeaways from these guidelines include: i) the importance of excluding ‘tic-like’ functional movement disorders before considering DBS; ii) the fact that reduction of tics should be the main objective of DBS aimed at tics, and thus the importance of ensuring that tics are the primary source of impairment; iii) the inclusion of patients who are refractory to pharmacologic and behavioral therapies; iv) the fact that DBS should be very cautiously considered in children and teenagers due to the frequent improvement of tics in adulthood; and v) the importance of performing DBS following a specified protocol within the context of controlled trials, cohort studies or registry databases. ^97^ These recommendations are in line with the opinion paper by Martino and colleagues, about the ‘five pillars’ for considering TS patients for DBS: i) high tic severity; ii) tic-related impact on quality of life; iii) treatment refractoriness; iv) stability of comorbid psychiatric disorders for six months; and v) multidisciplinary and particularly careful assessment of indications for patients under 18. ^98^

Two RCTs have been published which add to the data on efficacy of DBS in TS. Baldermann and colleagues published a trial involving eight patients with stimulation of the Cm-Voi nuclei of the thalamus with an interesting design of combined naturalistic open label with brief randomized, sham-controlled assessments. ^99^ They showed an improvement of tics with stimulation ON compared to sham stimulation, and an improvement in quality of life. Another study by Müller-Vahl and colleagues provided mixed findings. Their study involved 10 patients randomized to either sham, GPi, or thalamic stimulation. They found an improvement of tics with GPi stimulation and a superiority to GPi over thalamic and sham stimulation, with no overall improvement in quality of life, and with an important variability across patients. Importantly, four patients out of 10 had to be re-operated due to cable dysfunctions, and six years after surgery, five out of 10 patients had their stimulation turned off due to infections, technical problems, or lack of efficacy. ^100^ In an interesting clinical paper, MacLean *et al.,* report the complete resolution of GTS symptoms after the placement of stereo-EEG electrodes in the GPi and nucleus accumbens and suggest that stimulation at multiple patient-specific targets could provide effective control of GTS symptoms.This approach warrants further study. ^101^

Regarding the neural bases of DBS in GTS and the question of optimal stimulation target, several novel findings are worthy of attention. In a retrospective study involving 35 patients, Johnson and colleagues highlighted the important variability in pathway activation across patients and stimulation settings. ^102^ They showed a correlation between tic improvement and predicted activation of the associative pallido-subthalamic pathway, the ansa lenticularis, and the internal capsule tracts projecting to the prefrontal cortex. Anterior GPi stimulation may act via associative and limbic pathways modulation, whereas posterior GPi stimulation may have an effect on sensorimotor networks. Interestingly, Aminzade and colleagues reported the case of a patient with improvement of OCD, without improvement of tics, after anteromedial GPi stimulation. ^103^

A systematic review on clinical responses to several DBS targets in patients with TS showed fiber connectivity to be important in target selection because the effects of DBS on tics seem to occur through different networks at different stimulation sites. ^104^ Finally, an interesting case report described a patient with major depressive disorder who developed voltage-dependent coprolalia and tic-like movements along with changes in emotion, mood, and memory after DBS of bilateral ventral internal capsule and ventral striatum. ^105^

*Transcranial magnetic stimulation*

Focusing on the benefits of noninvasive brain stimulation as a potential treatment for TS, methodological (e.g., transcranial magnetic stimulation (TMS), transcranial direct current stimulation) and theoretical issues, ^106^ and TMS as a potential marker for TS ^107^ were discussed. Dual-site TMS and diffusion tensor imaging showed reduced prefrontal (pre-SMA) inhibition in children with TS/CTD compared to controls and the decreased inhibition correlated with impairment of tic suppressibility. ^108^ In addition, increased fractional anisotropy was observed in several white matter pathways in patients with TS/CTD, supporting a plausible pathophysiological mechanism associated with tic persistence.

Dyke *et al.,* present an exhaustive review of non-invasive brain stimulation in the treatment of TS. ^106^ One original study on low-frequency repetitive transcranial stimulation (rTMS) that stands out uses the bilateral parietal cortex as the target zone for the treatment of tics. The results, in 30 adult patients with TS, were very encouraging on all measures (YGTSS, MRVBTS and PUTS scores). Importantly, the beneficial effects lasted for up to a month after the last rTMS session. ^109^ This approach warrants replication and extension in larger cohorts. In children (n=10), bilateral rTMS of the supplementary motor area was well tolerated and decreased tic severity post-treatment. ^110^ However, this was an open label trial and requires confirmation in a blinded setting, and preferably over longer time periods.

*Other treatments*

The Movement Disorders Society Tourette Syndrome Study Group performed a Delphi survey amongst 36 international experts in 14 countries to better define the concept of treatment failure in persistent tic disorders, both with regard to pharmacological and behavioral therapy. ^111^ An operational definition was proposed, and its feasibility now needs to be assessed in clinical decision making, for instance in choosing candidates for DBS. The Movement Disorders Society Tourette Syndrome Study Group also published a survey on clinical practice patterns in tic disorders and concluded that tic disorders remain understudied beyond a scope of dedicated experts, and that diagnosis and treatment still requires improvement across the globe. ^112^

### Tics, family, and society

Several tic experts argued that, although TS is not a rare disease according to international classifications, it remains understudied and should therefore qualify for public health regulations that direct funding to treatment research for orphan or neglected diseases. ^113^ This is also reflected by an excellent review highlighting the scarcity of specialized TS clinics and dedicated experts. ^114^ This international group investigated health service delivery and care practices by clinicians in different geographical settings (Canada, US, Europe, and the United Kingdom). The results suggested that there is a scarcity of specialized TS clinical care in all investigated regions. Similarly, a study from the Tourette OCD Alberta Network reported on pitfalls and solutions for development of systemic care networks for tic detection. ^115^ In this mixed-methods study, 10 parents were interviewed in person and 140 parents responded to a survey. Qualitative data showed there was often an absence of a clear pathway to access healthcare for people with TS and OCD. Importantly, several solutions to tackle this problem were identified, such as: preparation of school-based training webinars, educational outreach in schools, and peer support.

*Quality of life*

Lee and colleagues ^116^ explored the role of self-esteem in mediating the relationship between psychosocial stress and social adjustment among adolescents with TS. Self-esteem of adolescents with TS fully mediated the relationship between psychosocial stress and social adjustment, while comorbidities moderated the relationship between self-esteem and social adjustment.

Solis-Garcia *et al.,* ^117^ studied quality of life and psychiatric comorbidities in pediatric patients with TS. In accordance with previous reports, higher severity of tics and ADHD were associated with poorer quality of life.

## Conclusions

The year 2021 saw publication of important work on a variety of fronts. Larger and collaborative studies produced ever more frequent, important contributions. Additionally, the flexibility of the TS clinical and research community was demonstrated by its response to the explosion of rapid-onset Tourette-like cases, beginning with rapid publication of case series but progressing quickly over the past one to two years to include international collaborations, controlled investigations, and prospective follow-up studies. Of perhaps greatest interest to our patients, new and actionable information appeared regarding numerous approaches to treatment of TS. These included the full range from psychological interventions to pharmaceuticals to non-invasive and invasive brain stimulation, and several clinical trials examined approaches that increased the availability of care at lower cost and to patients for whom frequent visits to a specialty center are difficult or impossible.

**Figure 1.**
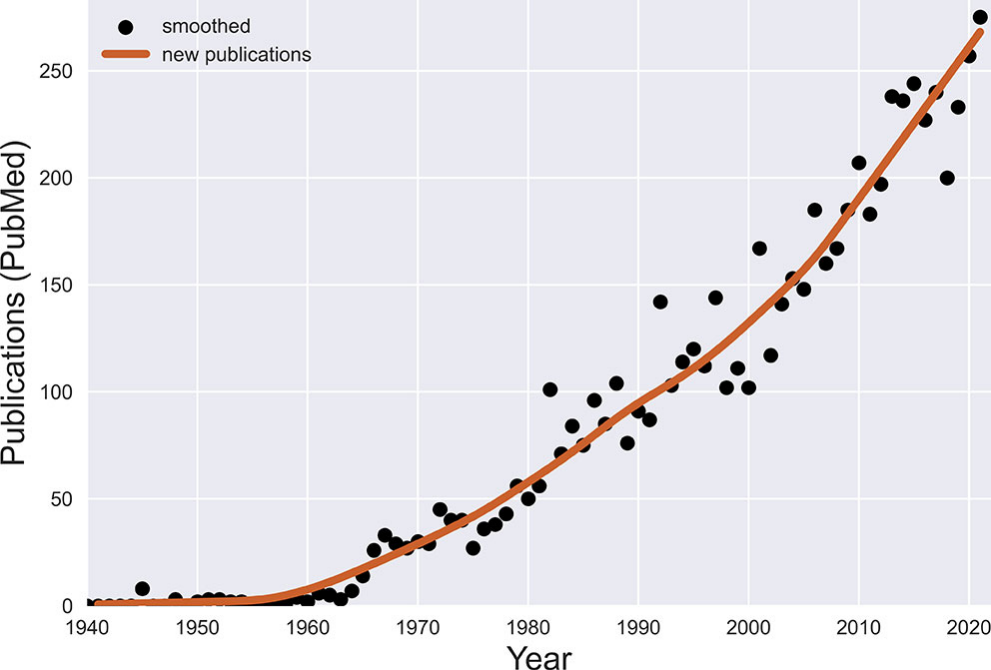
Publications on Tourette syndrome indexed annually by the (U.S.) National Library of Medicine. Image created by authors.

Keeping up with the relevant literature on TS has become increasingly difficult. Over the eight years since we began this yearly review, PubMed has indexed almost 2,000 new publications dealing with Tourette syndrome and other tic disorders, with a record 300 publications in 2021 (see Figure 1). Despite the length of this review, the authors suspect that we have omitted some important work or may have overstated the relevance of a publication mentioned above. To improve these reviews, we warmly invite readers both to comment on this article after publication and to nominate important articles from 2022 (including their own best work) by commenting on the draft for next year’s highlights article.
