# Supplementary material for: Tourette syndrome research highlights from 2021
Source: F1000Res. 2022 Nov 1;11:716. Originally published 2022 Jun 29. [Version 2] doi: 10.12688/f1000research.122708.2 (PMC9315233; doi:10.12688/f1000research.122708.2)
Supplement: Peer review history

# Peer review: Reviewer response for version 2

**DOI:** 10.5256/f1000research.138056.r154756

**Published:** 2022-11-14

**Authors:** Erica L. Greenberg

**Affiliations:** 1 Department of Psychiatry, Massachusetts General Hospital, Boston, MA, USA; 2 Harvard Medical School, Boston, MA, USA

Looks good!

 Few final thoughts

- Re the 'cognitive tics' - I would change the word anxiety to distress (assuming distress is accurate) because a key reason that OCD was separated from anxiety disorders is that one's obsessions do not have to be associated with anxiety per se (i.e. can also be disgust, distress).
- Re the FND paragraph - looks a lot better, however, there does still appear to be a circular logic component to the finding, even with it being prospective, as the authors are choosing rapid-onset as a proxy for FND (and that itself is not 'proven'). I'm not saying what is written is incorrect or that the differences between the groups aren't true, but I would be careful about saying it's not circular since the authors are subjectively picking the criteria they are using to differentiate the groups.

Is the review written in accessible language?

Yes

Are all factual statements correct and adequately supported by citations?

Partly

Are the conclusions drawn appropriate in the context of the current research literature?

Yes

Is the topic of the review discussed comprehensively in the context of the current literature?

Yes

Reviewer Expertise:

Tourette syndrome and co-occurring related disorders, including OCD. Child and adolescent psychiatry.

I confirm that I have read this submission and believe that I have an appropriate level of expertise to confirm that it is of an acceptable scientific standard.

# Peer review: Reviewer response for version 1

**DOI:** 10.5256/f1000research.134731.r142616

**Published:** 2022-07-25

**Authors:** Erica L. Greenberg

**Affiliations:** 1 Department of Psychiatry, Massachusetts General Hospital, Boston, MA, USA; 2 Harvard Medical School, Boston, MA, USA

Thank you for the opportunity to review this comprehensive review of the 2021 literature covering Tourette syndrome (TS) and related disorders. The authors did a wonderful job presenting a thorough, detailed, yet succinct summary of an enormous number of new, important articles in the field. I appreciated that they showed the growth of research in TS over the last forty years, and are open to creative ways to make sure they have access to the likely even larger 2022 literature base. They covered a range of topics, broken down in effective sections, including Phenomenology and Natural History, Pathophysiology, Electrophysiology, Treatment, each with its own appropriate subsections. The authors presented the brief summaries of the literature clearly, and without bias. 

 I only have a few comments/recommended adjustments that I would suggest the authors consider following up on.

- Under Epidemiology, starting " *The controversial topic of cognitive...*" - it would help if the authors add a sentence clarifying why that phenomena is not considered OCD/intrusive thoughts.
- Under Comorbidity, starting " *A very interesting study...*" - I would recommend either eliminating that or expanding on it, as the findings are quite provocative, and given the sample size was so small, I worry about it being given more attention than warranted without further studies to validate (and/or without commenting on co-occurring conditions that may also influence moral reasoning).
- Under Premonitory Urge and Tic Suppression, starting " *Another study, by He...*" - I would make that a new paragraph
- Under Environmental Risk Factors, starting " *This year brought,*" I would recommend condensing the paragraph to make it more in line with the other summaries, particularly related to citations 46 and 47. Given that they were small studies, particularly 47, I would recommend not listing all the findings/differences (particularly as I wonder about multiple comparisons in that study..). Related, I would soften the sentence saying "meaning that almost none of these patients' presentations even slightly resembled TS to these clinicians" as it sounds more subjective/intense than the rest of the paper. And I would soften the language in the paragraph starting " *One concern...*" as again, it read as more subjective/opinion-driven than the rest of the manuscript.
- In the paragraph starting, " *Sturm and colleagues...*" I believe the heading above it is incorrect.
- Under Tic Assessment, starting " *Many studies have focused...*" it would help to clarify the last sentence starting, " *Interesting vocal tics...*" Without more context, it is difficult to understand why vocal tics not predicting TS diagnosis might challenge the validity of diagnostic TS criteria vs challenge the validity of the SVM (support vector machine)?
- Under Treatment Psychological interventions, starting " *Several research groups...*" - it would help to spell out what the acronym BIP means
- In the figure depicting the increase in TS related literature over time, it looks like the legend is flipped
- In Conclusions, I wonder if the authors mean to say "Additionally" rather than " *On the other hand.*"

Is the review written in accessible language?

Yes

Are all factual statements correct and adequately supported by citations?

Partly

Are the conclusions drawn appropriate in the context of the current research literature?

Yes

Is the topic of the review discussed comprehensively in the context of the current literature?

Yes

Reviewer Expertise:

Tourette syndrome and co-occurring related disorders, including OCD. Child and adolescent psychiatry.

I confirm that I have read this submission and believe that I have an appropriate level of expertise to confirm that it is of an acceptable scientific standard.

# Response

**Published:** 2022-08-29

**Authors:** Andreas Hartmann

**Affiliations:** Pitié-Salpêtrière Hospital, Paris, France

We thank Dr Greenberg for her positive evaluation of our manuscript and her helpful comments. We have incorporated nearly all comments except the passages on FND which we did shorten only marginally. Because many readers will not be familiar with the clinical presentation of these patients, a bit more detail seemed appropriate to us even if the case numbers were small. However, we agree that in the absence of larger studies and harder data (coming up in 2022, hopefully!), much is opinion-driven at this stage. Also, we do not know what the BIP in BIP TIC stands for, it is an abbreviation that has been used by the Karolinska group from the beginning and might actually be Swedish. Finally, we believe the figure legend to be adequate.

# Peer review: Reviewer response for version 1

**DOI:** 10.5256/f1000research.134731.r142613

**Published:** 2022-07-18

**Authors:** Daniel Alvarez-Fischer

**Affiliations:** 1 Institute of Systems Motor Science, University of Lübeck, Lübeck, Germany

The authors provide a good overview of the current literature. This annual summary of the current scientific literature is an important milestone for people active in Tourette's research and treatment to keep themselves informed.

 The selection of literature is presented clearly and understandably. Perhaps the categories into which the selected literature is placed could also be presented in an overview at the beginning. Such a list would make reading even more straightforward. The authors mention that they have also integrated congress contributions. A list of the selected congresses would be desirable.

 There is an interesting graph at the end of the review about the increasing number of annual publications in the field. Probably the number of publications is still too small; if not, a map from which countries how many publications come, and from which countries how many publications are taken into account (e.g., by the size and color of dots on a map) would be an interesting, but not necessary information for the publication.

 The present manuscript is a very successful work that merits indexing in this form.

Is the review written in accessible language?

Yes

Are all factual statements correct and adequately supported by citations?

Yes

Are the conclusions drawn appropriate in the context of the current research literature?

Yes

Is the topic of the review discussed comprehensively in the context of the current literature?

Yes

Reviewer Expertise:

Tourette's, Autism, and ADHD

I confirm that I have read this submission and believe that I have an appropriate level of expertise to confirm that it is of an acceptable scientific standard.

# Response

**Published:** 2022-08-29

**Authors:** Andreas Hartmann

**Affiliations:** Pitié-Salpêtrière Hospital, Paris, France

We thank Dr Alvarez-Fischer for his kind comments on our manuscript. The figure was a labor of love by Dr Black and adding geographic information is beyond what we can offer, at least this year. We agree, however, that this information might be of interest!
